# Disseminated Histiocytic Sarcoma in a Japanese Macaque ( *Macaca fuscata* )

**DOI:** 10.1111/jmp.70028

**Published:** 2025-08-01

**Authors:** Minami Sato, Haruka Takimoto, Yuki Matsubara, Tsunenori Tsujimoto, Jun Sasaki

**Affiliations:** ^1^ Department of Veterinary Pathology School of Veterinary Medicine, Iwate University Morioka Japan; ^2^ Morioka Zoological Park ZOOMO Morioka Japan

**Keywords:** abdominal cavity, histiocyte, immunohistochemistry

## Abstract

Histiocytic sarcomas are rare in humans and primates. Particularly, only a few examples in nonhuman primates have been reported. This is the first report of histiocytic sarcoma in *Macaca fuscata* . The final diagnosis was made using immunohistochemical analysis.

## 1 Introduction

Histiocytic sarcomas (HS) originate from histiocytes and are rare in humans. HS expands rapidly into internal organs, and the median survival time of HS is < 1 year [1]. In nonhuman primates, HS is an extremely rare and aggressive tumor. To date, HS has been reported in *Saimiri sciureus* , Pongo sp., common squirrel monkeys, slow loris, and cynomolgus macaques, but not in Japanese macaques ( *Macaca fuscata* ) [2, 3, 4, 5, 6, 7].

Neoplastic cells of HS have various cellular morphologies that incur diagnostic errors. Immunohistochemical staining is useful for definitive diagnosis in humans. Particularly, antibodies against Iba‐1 indicate high sensitivity [8]. However, HS cases that have been immunohistochemically stained in primates are rare; therefore, useful antibodies are uncertain.

This case report describes the histological features and phenotypes of Japanese macaques by using immunohistochemistry (IHC).

## 2 Case Report

The authors confirm that the ethical policies of the journal have been adhered to, as noted on the journal's author guidelines page. Ethical approval was not required as no animals were used in this study.

A 24‐year‐old female Japanese macaque weighing 5.5 kg was born and raised in Morioka Zoological Park ZOOMO, Japan. The animal suddenly exhibited recumbency. A physical examination revealed hypothermia. The animal had bloody diarrhea and could not move except for the fingers and toes. The blood count revealed increased levels of hematocrit value (56%). Biochemical analysis revealed decreased total protein (5.0 g/dL) and albumin (2.5 g/dL) levels. Despite treatment with antibiotics, hemostats, and steroids, the macaque died and necropsy was conducted.

Macroscopically, a large sized (approximately 9.7 × 10.4 × 7.0 cm) mass was found in the abdominal cavity invading into the thoracic cavity. On sectioning, the mass was pale yellow, solid, and multilobular. Similar multilobular masses were found in the spleen, lungs, omentum majus, mesentery, ovaries, and uterus. Atelectasis was observed in the right lung. Furthermore, the right side of the heart was dilated and several lymph nodes were swollen. The major organs, abdominal mass, omentum majus, ovaries, and uterus were fixed in 10% neutral phosphate‐buffered formalin. These samples were embedded in paraffin and stained with hematoxylin and eosin (H and E).

**FIGURE 1:**
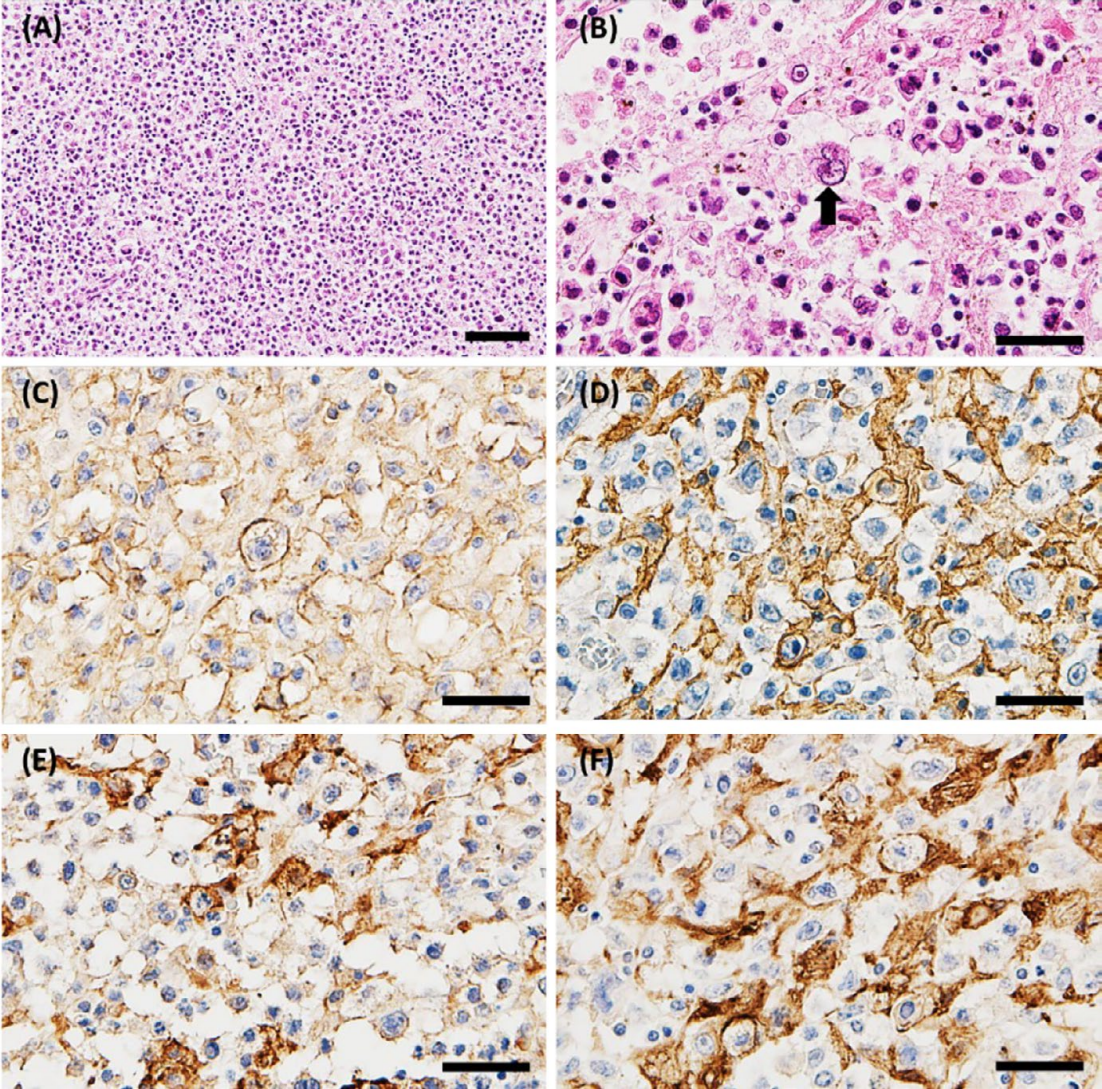
Histopathology in the neoplastic tissue. (A) Solid proliferation of neoplastic cells. (B) Neoplastic cells have round‐to‐oval or cleaved nuclei, and acidophilic abundant cytoplasm. (C–F) Neoplastic cells are positive for HLA‐DR (C), Iba‐1 (D), CD204 (E), and CD68 (F). (A, B) H and E stain. (C–F) IHC counterstained with hematoxylin. Bars, 50 μm (A), 20 μm (B–F).

Microscopically, the large abdominal mass had a fibrous capsule and comprised solid proliferations of polygonal to oval cell‐like histiocytes of various sizes (Figure 1A). The neoplastic cells had round‐to‐oval and sometimes cleaved nuclei and abundant acidophilic cytoplasm (Figure 1B). Multinucleated giant cells with large atypical nuclei were often observed. Round cells, such as lymphocytes, were combined with the neoplastic tissue. Severe postmortem changes were observed in most neoplastic areas. There were nodules, including neoplastic cells, such as histiocytes, on the splenic capsule. The white pulp showed mild atrophy. In the lungs, neoplastic cells proliferated in the stroma and invaded lymphatic vessels. The parenchyma of the ovary, uterus, and omentum was almost completely replaced by the neoplastic tissue.

IHC was performed on ovary sections. The primary antibodies and results are listed in Table 1. The neoplastic cells were positive for vimentin, lysozyme, ionized calcium‐binding adapter molecule 1 (Iba‐1), CD68, CD204, and human leukocyte antigen‐DR (HLA‐DR) (Figure 1C–F). There was weakly positive labeling for Ki‐67 and negative labeling for CD3 and BLA36. The tumor cells were negative for peroxidase staining.

**TABLE 1:** Primary antibody for immunohistochemistry.

Antigen	Source	Clone	Dilution	Result
Vimentin	Agilent	V9	1:50	+
Lysozyme	Agilent, Denmark	Polyclonal	1:1000	+
Iba‐1	Wako	Polyclonal	1:50	+
CD68	antibodies‐online.com	Y1/82A	1:800	+
CD204	Agilent, Denmark	SRA‐E5	1:800	+
HLA‐DR	Agilent	TAL 1B5	1:400	+
Ki‐67	Agilent	SolA15	1:100	±
CD3	Agilent, Denmark	Polyclonal	1:50	NS
BLA36	Thermo	EBS‐T‐002	Ready‐to‐use	+
AE1/AE3	Invitrogen, U.S.A	Cytokeratin	Ready‐to‐use	—

Abbreviations: HLA‐DR, human leukocyte antigen‐DR; Iba‐1, ionized calcium‐binding adapter molecule 1.

## 3 Discussion

This is the first report of HS in the Japanese Macaque. Neoplastic tissues were observed in the spleen, lungs, and ovaries. This finding revealed that the tumor had metastasized systemically. Previous HS cases in primates and this case occurred in the body cavity. In humans, HS commonly arises in the intracavitary organs and lymph nodes [9]. The HS in dogs occur in the joints, lymph nodes, liver, and spleen. The lesions are sometimes extensively formed in the abdominal and thoracic cavities (disseminated HS) [10]. In dogs with disseminated HS, mutations in the PTPN11 gene were found [11]. This mutation is commonly noted in juvenile myelomonocytic leukemia in human [12]. Moreover, it is found in humans with HS [13]. PTPN11 may be involved in the carcinogenesis of HS in other primates. IHC is often performed for the definitive diagnosis of HS in humans and dogs. There is a standard for the effective antibodies used for diagnosis in humans. The neoplastic cells of human HS show a positive reaction to CD68, CD163, Iba‐1, CD4, and lysozyme [14]. In dogs, CD18, Iba‐1, HLA‐DR, and CD204 are known to be highly sensitive antibodies [15]. HLA‐DR and Iba‐1 were positive in all cases of the tested non‐human primate HS. However, HLA‐DR reacts with lymphocytes; thus, it is perceived as a less specific antibody [13]. Conversely, Iba‐1 is a specific antibody to the histiocytic system [8]. Hence, Iba‐1 may be an effective antibody for diagnosing HS in non‐human primates. In the present case, peroxidase staining was negative for tumor cells, indicating a tumor of non‐myeloid origin.

Currently, only two cases of HS expressing a positive reaction to CD204 have been reported in non‐human primates [5]. CD204 is a class A macrophage scavenger receptor. IHC for CD204 was reportedly positive in all cases of HS in dogs [16]. Therefore, this antibody may be highly sensitive. However, immunostaining for CD204 has only been performed in a few cases of HS in non‐human primates; therefore, additional data are needed to assess its diagnostic efficacy. Additionally, there was only one case of immunostaining for CD68 in non‐human primate HS [7]. However, the case showed the same positive reaction as in the present case. Therefore, CD68 may be an effective marker for diagnosing HS in non‐human primates.

In humans, the differential diagnosis of HS also includes follicular dendritic cell sarcoma (FDCS). To the best of our knowledge, no previous study has addressed the differential diagnosis of these two neoplasms in non‐human primates. CD68 is commonly used as a histiocytic marker and is typically positive in HS; however, its expression in human FDCS has been reported to be variable [17]. Furthermore, there have been no reported cases of CD204 positivity in human FDCS, which supports the exclusion of FDCS in this case.

In conclusion, we suggest that Iba‐1 is a useful antibody for diagnosing HS of non‐human primates in this study. Moreover, CD204 and CD68 may be helpful. However, there were only a few cases of IHC; therefore, we could not determine the phenotype of HS in non‐human primates. IHC using several antibodies is necessary to diagnose HS.

## Conflicts of Interest

The authors declare no conflicts of interest.

## Data Availability

The data that support the findings of this study are available from the corresponding author upon reasonable request.

## References

[1] E. Ralfkiaer , G. Delsol , N. T. O'Connor , et al., “Malignant Lymphomas of True Histiocytic Origin. A Clinical, Histological, Immunophenotypic and Genotypic Study,” Journal of Pathology 160 (1990): 9–17, 10.1002/path.1711600105. 2156039

[2] A. Buchanan , J. Díaz‐Delgado , G. Balamayooran , M. Anguiano , K. Groch , and L. Krol , “Leukemic Histiocytic Sarcoma in a Captive Common Squirrel Monkey (*Saimiri Sciureus*) With Saimiriine Gammaherpesvirus 2 (Rhadinovirus), *Saimiri Sciureus* Lymphocryptovirus 2 (Lymphocryptovirus) and Squirrel Monkey Retrovirus (β‐Retrovirus) Coinfection,” Journal of Medical Primatology 49 (2020): 341–343, 10.1111/jmp.12471. 32412106

[3] D. L. Sly , D. H. Ringler , and M. R. Anver , “Spontaneous Malignant Histiocytoma With Metastasis in a Rhesus Monkey (*Macaca Mulatta*),” Journal of Medical Primatology 6 (1977): 43–49, 10.1159/000459715. 194039

[4] M. Canuti , C. V. Williams , S. R. Gadi , et al., “Persistent Viremia by a Novel Parvovirus in a Slow Loris (*Nycticebus Coucang*) With Diffuse Histiocytic Sarcoma,” Frontiers in Microbiology 5 (2014): 655, 10.3389/fmicb.2014.00655. 25520709 PMC4249460

[5] M. Sakurai , Y. Yamamoto , M. Tamaru , H. Shimoda , Y. Sakai , and M. Morimoto , “Disseminated Histiocytic Sarcoma in a Squirrel Monkey (*Saimiri Sciureus*),” Journal of Medical Primatology 52 (2023): 121–124, 10.1111/jmp.12625. 36286409

[6] V. Galietta , N. Fonti , C. Cocumelli , et al., “Histiocytic Sarcoma in a Captive Hybrid Orangutan (Pongo sp.): Morphological and Immunohistochemical Features,” Animals 14 (2024): 852, 10.3390/ani14060852. 38539950 PMC10967491

[7] T. Soshin , K. Adachi , S. Suzuki , et al., “Histiocytic Sarcoma in a Cynomolgus Macaque ( *Macaca fascicularis* ) Fed With a High‐Fat Diet,” J Toxcal Pathol, no. 21 (2008): 69–72, 10.1293/tox.21.69.

[8] X. Zhang , L. P. Wang , A. Ziober , P. J. Zhang , and A. Bagg , “Ionized Calcium Binding Adaptor Molecule 1 (IBA1),” American Journal of Clinical Pathology 156 (2021): 86–99, 10.1093/ajcp/aqaa209. 33582751

[9] A. Kommalapati , S. H. Tella , M. Durkin , R. S. Go , and G. Goyal , “Histiocytic Sarcoma: A Population‐Based Analysis of Incidence, Demographic Disparities, and Long‐Term,” Blood 131 (2018): 265–268, 10.1182/blood-2017-10-812495. 29183888 PMC5757688

[10] C. Mullin and C. A. Clifford , “Histiocytic Sarcoma and Hemangiosarcoma Update,” Veterinary Clinics of North America. Small Animal Practice 49 (2019): 855–879. 31186126 10.1016/j.cvsm.2019.04.009

[11] M. Takada , L. A. Smyth , T. Thaiwong , et al., “Activating Mutations in PTPN11 and KRAS in Canine Histiocytic Sarcomas,” Genes 10, no. 7 (2019): 505, 10.3390/genes10070505. 31277422 PMC6678586

[12] M. Tartaglia , C. M. Niemeyer , A. Fragale , et al., “Somatic Mutations in PTPN11 in Juvenile Myelomonocytic Leukemia, Myelodysplastic Syndromes and Acute Myeloid Leukemia,” Nature Genetics 34 (2003): 148–150, 10.1038/ng1156. 12717436

[13] B. Hédan , M. Rault , J. Abadie , et al., “PTPN11 Mutations in Canine and Human Disseminated Histiocytic Sarcoma,” International Journal of Cancer 147 (2020): 1657–1665, 10.1002/ijc.32991. 32212266

[14] S. H. Swerdlow , E. Campo , N. L. Harris , et al., World Health Organization Classification of Tumors. Pathology and Genetics of Tumors of Haematopoietic and Lymphoid Tissues, 4th ed. (IARC Press, 2008), 356–357.

[15] T. Ide , K. Uchida , Y. Kagawa , K. Suzuki , and H. Nakayama , “Pathological and Immunohistochemical Features of Subdural Histiocytic Sarcomas in 15 Dogs,” Journal of Veterinary Diagnostic Investigation 23 (2011): 127–132, 10.1177/104063871102300123. 21217043

[16] Y. Kato , M. Murakami , Y. Hoshino , et al., “The Class A Macrophage Scavenger Receptor CD204 Is a Useful Immunohistochemical Marker of Canine Histiocytic Sarcoma,” Journal of Comparative Pathology 148 (2013): 188–196, 10.1016/j.jcpa.2012.06.009. 22901707

[17] S. L. Skala , D. R. Lucas , and R. Dewar , “Histiocytic Sarcoma: Review, Discussion of Transformation From B‐Cell Lymphoma, and Differential Diagnosis,” Archives of Pathology & Laboratory Medicine 142 (2018): 1322–1329. 30407858 10.5858/arpa.2018-0220-RA

